# Indicator-driven data calibration of expert interviews in a configurational study

**DOI:** 10.1016/j.mex.2022.101699

**Published:** 2022-04-26

**Authors:** Henriette Naims, Elisabeth Eppinger

**Affiliations:** a Freie Universität Berlin, School of Business and Economics, Thielallee 73, Berlin 14195, Germany; b Institute for Advanced Sustainability Studies e.V., Berliner Str. 130, Potsdam 14467, Germany; c Hochschule für Technik und Wirtschaft Berlin, Wilhelminenhofstraße 75A, Berlin 12459, Germany

**Keywords:** fsQCA, Data calibration, Expert interviews, CCU

## Abstract

Expert interviews can provide interesting data for the use in qualitative comparative analysis (QCA) to investigate complex social phenomena. To guide the challenging task of data calibration from qualitative data sets, techniques have already been suggested for the transformation of qualitative data into fuzzy sets. The current article follows existing guidelines and extends them with a system for indicator-based data calibration of expert interviews. While the underlying data set is confidential due to its corporate setting, in this article the analysis of the data is made transparent and hence reproducible for potential follow-up studies. First, the process of data collection is described, and the final data sample is characterized. Consequently, a system for indicator-based data calibration is presented and the calibration results for the empirical sample are provided in form of the set membership of cases and truth tables.

• Data collection from expert interviews is described for a configurational setting

• A combined indicator-based system is used for the calibration of qualitative data

## Graphical abstract

**Figure:**
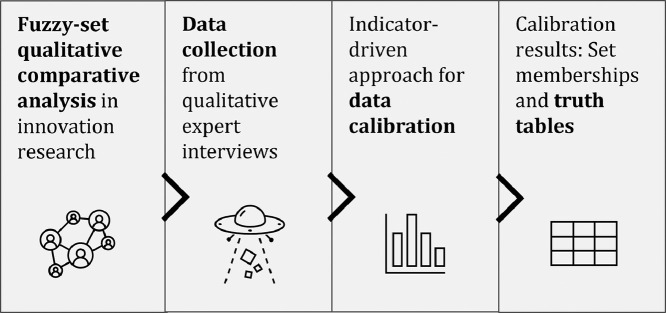

Specifications Table

**Table:** 

Subject Area;	*Economics and Finance*
More specific subject area;	*Business research*
Method name;	*Data calibration for fsQCA*
Name and reference of original method;	• *Ragin, C. C.*[1]*. Redesigning social inquiry: Fuzzy sets and beyond. Chicago/London: University of Chicago Press.* • *Basurto, X., & Speer, J.*[2]*. 'Structuring the calibration of qualitative data as sets for qualitative comparative analysis (QCA)'. Field Methods, 24(2), 155-174. doi:10.1177/1525822×11433998* • *De Block, D., & Vis, B.*[3]*. 'Addressing the challenges related to transforming qualitative into quantitative data in qualitative comparative analysis'. Journal of mixed methods research, 13(4), 503-535. doi:10.1177/1558689818770061*
Resource availability;	*The analysis is based on confidential primary data from interviews with corporate innovation managers that cannot be shared. The interview data was verified and triangulated via additional quantitative and qualitative data from participatory observations at conferences and desk-based research into the corporations and their activities.*

## Method details

### Fuzzy set qualitative comparative analysis (fsQCA)

Qualitative comparative analysis (QCA) is a well-established field of methods to investigate causal configurations, originally developed and further refined by Ragin [1,^4^,5]. QCA enables causal investigations into social phenomena based on set theory and qualitative or quantitative information and has found wide application across research fields at the macro, meso, and micro levels. For example, in innovation research several studies have recently applied QCA with a focus on innovation systems [6,7], innovation clusters [8], innovation performance [9], [10], [11], management innovations [12], service innovations [13,14] and eco-innovations [15]. An important advantage compared to conventional methods is that QCA allows for equifinality, i.e., more than one causal path to an outcome [16]. Moreover, causality is directional and one-way and hence can be asymmetric [16]. For management and business research, Misangyi et al. [17] claim that QCA enables a “neo-configurational perspective” that is particularly promising for certain types of research, including studies on expected but unobserved strategy, strategic change, and managerial decision making.

Hence, the present study conducts a QCA according to Ragin [1] and the best practices for strategy and organizational research defined by Greckhamer et al. [18] to investigate unobserved strategies among firms involved in research and development (R&D) for carbon capture and utilization (CCU) technologies. While QCA can be applied to different kinds of sets, fuzzy sets allow researchers to calibrate their data between non-membership (0.0) and full membership (1.0) in ordinal or continuous scales based on substantial knowledge and qualitative assessment [1,5]. Due to the complexity of the task, this article presents in detail the data collection process from expert interviews and the subsequent data calibration for the original research article [19]. Since data calibration is of paramount importance for the quality of QCA, this article follows the research techniques suggested by Basurto and Speer [2] and De Block and Vis [3] and further details an indicator-driven approach for data calibration in the specific research context.

## Data collection

### Preparation of the interview guidelines

To prepare data collection, as a first step a guideline for the interviews with innovation managers (see Table 1) was prepared incorporating epistemological and methodological recommendations for expert interviews from Bogner et al. [20] and the theoretical concepts of a configurational system of innovations (see Naims and Eppinger [19]). The guideline starts out with the collection of relevant personal information (section A) and company information (section B). Section C presents a definition of CCU and facilitates a discussion to reach a common understanding or uncover potential discrepancies between the interviewer and the interviewed expert. Section D collects all relevant information on the status of the CCU projects within the firm. The subsequent sections were designed to harvest the experts’ knowledge of R&D resources, results, policy conditions, and their expectations for economic progress: Section E collects information on profitability and production costs, section F on revenues, section G on intangible value, section H on Investments, section I on policy and external conditions, and section K on economic progress. The list of criteria and questions were formulated based on the research targets and the theoretical literature, in particular Grupp [21].

**Table 1:** Guideline for expert interviews.

Section	Topic	Data source
A	Personal information	
	Department	interview
	Region of responsibility	interview
	Work experience within industry in years	interview
B	Company information	
	Major products /sector (ISIC code)	Annual Report
	Revenues 2017 in m US$	Annual Report
	R&D Investments 2017 in m US$	Annual Report
	Reported CO_2_ emissions 2017 (Scope 1+2 in Mt)	Annual Report
	Employees 2017	Annual Report
	Firm size	Annual Report
C	Definition of CCU	
	Definition: Carbon capture and Utilization comprises both industrial capture to obtain concentrated CO_2_, and separate functional utilization of this CO_2_. In general, the following three different utilization options are differentiated: - Direct utilization, i.e., using the carbon dioxide itself without a transformation, for example in carbonated beverages or food packaging. - Utilization in materials, i.e., conversion to carbon-based chemical products such as plastics and foams. - Utilization in energy carriers, i.e., conversion to hydrocarbon fuels such as methanol and synthetic gas.	interview
D	Status of CCU activities	
	Which CCU-based product(s) does the firm have?	interview
	In which CCU research projects is the firm active?	interview
	How long has the firm been active in CCU?	interview
	What was the trigger for starting CCU?	interview
	What was the motivation for starting CCU?	interview
	What is the TRL level of the CCU activities?	interview
E	Profitability & Production Costs	
	How will production costs be affected by the introduction of CCU (compared to conventional technologies)?	interview
	How are production inputs impacted by the CCU innovation?	interview
	Where does the CO_2_ for the CCU process come from?	interview
	What is the price of CO_2_?	interview
	Which role will transport costs of CO_2_ play?	interview
	Is there a profit margin on CO_2_? Which one?	interview
	What is the effect on efficiency of the production process?	interview
	How will the profit margin of the CCU based product change (compared to conventional technologies)?	interview
	What structural effects on the firm's suppliers do you expect?	interview
F	Revenues	
	How will revenues be affected by the introduction of CCU (compared to conventional technologies)?	interview
	Has an LCA been conducted for the CCU process? What is the result?	interview
	To what extent does the CCU-based product have different characteristics?	interview
	Will the CCU product replace/compete with existing products?	interview
	Will the CCU product extend the product portfolio?	interview
	Will the CCU product will be introduced to an existing market?	interview
	Will the CCU product be introduced to a new market?	interview
	To what extend will the production of the conventional product change?	interview
	Will there be a price difference compared to conventional products? Which one?	interview
	To what extent are revenues from IP licensing planned?	interview
	What structural effects on the firm's customers do you expect?	interview
G	Intangible Value	
	How many patents have been submitted/granted in CCU?	interview
	How will the public image of the products be affected by CCU?	interview
	How important is the public image of the product to the firm?	interview
	Does the firm measure the public image of product?	interview
	CCU is expected to improve customer satisfaction.	interview
	How important is customer satisfaction to the firm?	interview
	Does the firm measure customer satisfaction?	interview
	Does the firm have a sustainability strategy?	interview
	Does the firm have a sustainability reporting in place?	interview
	How important is sustainability reporting to the firm?	interview
	To what extend has CCU been communicated in the context of sustainability reporting?	interview
	Is CCU expected to improve public relations of the company?	interview
	How important are public relations to the firm?	interview
	To what extent have stakeholders shown interest in the CCU activities?	interview
	What were the reactions of the stakeholders?	interview
H	Investments	
	How much has been invested into R&D of CCU at which plants? (Total, Capex, Opex)	interview
	When and where have these investments taken place?	interview
	How much external funding has the firm received for CCU?	interview
	How important is the acquisition of external funding for CCU?	interview
	Would you have pursued CCU without external funding?	interview
	How many people have been/are working on CCU?	interview
	What is the background of these people?	interview
I	Policy & external conditions	
	What are potential external barriers to CCU development?	interview
	What role does the EU ETS play for CCU and why?	interview
	Which regulations/policies/standards can play a role for CCU development? Which one?	interview
	Which major market trends do you observe and how to they combine with CCU?	interview
K.	Economic progress	
	To what extent will CCU have employment effects? For whom?	interview
	To what extend can CCU affect or trigger economic growth? For whom?	interview
	To what extent can CCU strengthen or harm a local industry?	interview
	Do you observe new firms, products, funding from CCU (entrepreneurship)?	interview
	To what extend can CCU modernize the industry?	interview
	To what extend allows CCU for synergies in the value chain (industrial symbiosis, sector integration)?	interview
	Which other economic impacts do you expect?	interview
	Which topic has been missing? What would you like to add?	interview

### Expert selection and interview process

Initial candidates for the expert interviews were representatives of firms identified from the participant lists and agendas of relevant scientific or business conferences and workshops on CCU (see Table 2) in which the authors participated between 2014 and 2017. Upon invitation, experts from only three companies declined to participate. Thus, the final sample sufficiently represents the available expertise on CCU in European-based corporations that actively (and publicly) engaged in CCU development at the time. Representatives of firms that initiated R&D activities on CCU more recently could not be included. While incremental progress is possible, a significant technological advancement since the interviews seems unlikely due to the commonly long timeframes of around 10 years from the decision to invest to start of operations (Bazzanella & Ausfelder, 2017).

**Table 2:** List of conferences and workshops on CCU.

Event name	Date	Location
Acatech Workshop: Technische Wege/Pfade zur Dekarbonisierung	February 21–22, 2017	Berlin, Germany
I-SUP 2016: CCUS Carbon Capture & Utilisation	October 19, 2016	Antwerp, Belgium
14th International Conference on Carbon Dioxide Utilisation (ICCDU)	September 12–15, 2016	Sheffield, UK
SCOT Final Conference: CO_2_ Utilisation as a Catalyst for the European Industrial Renaissance	June 29, 2016	Brussels, Belgium
5th Carbon Dioxide Utilisation Summit	October 21–22, 2015	Dresden, Germany
Gordon Research Conference: Carbon Capture, Utilization & Storage	May 31–June 5, 2015	Easton, MA, USA
5. BMBF Status Conference “Technologies for Sustainability and Climate Protection – Chemical Processes and Use of CO_2_”	April 21–22, 2015	Berlin, Germany
CO_2_ Forum	September 24–26, 2014	Lyon, France

Each interview was prepared in advance based on participatory observations at the listed conferences (Table 2) combined with desk-based research into the corporations and their CCU-related activities, their intangible assets, and economic performance. When analyzing the interviews, further questions could be addressed to the interviewees. The data collected in the interviews hence could be sufficiently verified and triangulated.

In a few cases, two experts were interviewed per company. Then, certain parts of the interview were split between interviewees depending on their respective knowledge and corporate functions; for example, a marketing expert answered the marketability questions while an R&D expert answered the investment questions. Since these experts always work in project teams, in those cases the individual responses of the separated sections were treated as valid for both experts. However, in other cases, two experts from the same company completed the entire interview, for example when both worked in R&D but in different business units. The section on economic progress was completed by all experts individually in order to cover the entire qualitative spectrum of their expectations for achieving growth and transformation goals based on their personal and context-specific experience and beliefs. We conducted the in-depth semi-structured interviews in person, or by video/phone call in German or English between 06/2016 and 03/2017. The interviews lasted between one to three hours. The dialogue was recorded and consequently transcribed.

### Characterization of the final sample and the data

**Fig. 1:**
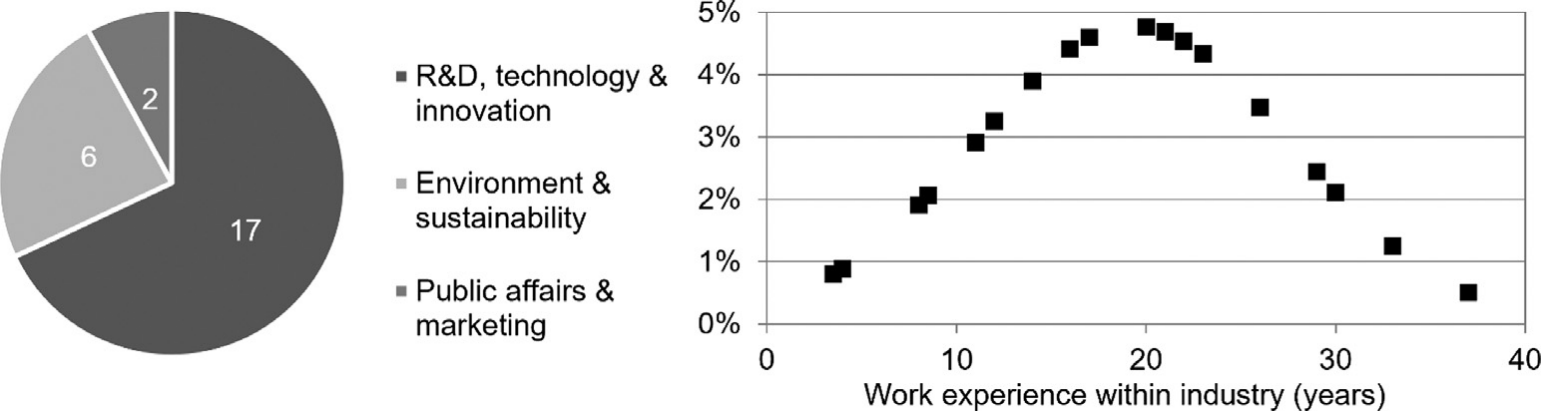
Characterization of experts by departments and work experience. Sources: Expert interviews.

The interviewed experts are, overall, highly experienced professionals who serve diverse departments, including R&D, technology and innovation, environment and sustainability, public affairs, and marketing (see Figure 1).

The interviews examined the experts’ knowledge of R&D resources, results, policy conditions, and their expectations for economic progress. Since all interviewees are involved in the management and/or advancement of corporate R&D projects, their knowledge of R&D resources and results is of very high quality. Furthermore, most experts are very knowledgeable about policy conditions relevant for R&D in CCU. While those experts from public relations or environmental departments often have a more detailed knowledge of policies, even those with a more technical R&D background were able to reflect on the marketability conditions of their work in detail. Despite their subjectivity, all expectations are shaped within a profit-driven environment with explicit or implicit innovation strategies. Hence, the experts’ expectations provide valuable insights on the progress potentials of such innovations.

Moreover, we analyzed public company data for the experts’ firms to further characterize the sample. For this, the CO_2_ intensity of the firm was calculated as the ratio of CO_2_ emissions (including scope 1 and scope 2) to revenues measured in tCO_2_/m US$. This measure is common for environmental, social, and governance (ESG) stock market index evaluations, e.g., MSCI Inc. [22]. Intervals were classified based on the observed distribution of the sample as low when below 300, as medium when between 300 and 800, and as high when above 800. Moreover, the R&D intensity of the firm was calculated as the ratio of R&D expenses to revenues. Intervals were classified as follows: low is below 1%, medium is between 1% and 4 %, and high is above 4%. This is in line with the classification by the European Commission [23] except that the latter defines high R&D intensity as above 5%. In contrast, Grupp [21] defines high R&D intensity as above 3.5%. Since our sample only contains one firm with an R&D intensity between 3.5% and 5%, this was categorized as high, and the corresponding boundary set to values above 4%. Data on revenues, R&D expenses, and CO_2_ emissions (including scope 1 and scope 2) is sourced from annual reports for the year 2017. Only for one start-up company, financial data for 2017 were unavailable and replaced by data for 2018. For two start-ups, emissions data were unavailable but were assumed to be in the low category. The analysis of the firms reveals three groups: (i) CO_2_-intensive firms with low R&D intensity, (ii) R&D-intensive firms with low CO_2_ intensity, and (iii) firms with medium CO_2_ intensity and low or medium R&D intensity (see Naims and Eppinger [19]).

The presented attributes characterize the sample. Due to its small size systematic sensitivities of the result towards certain characteristics of the expert (background, experience) or the firm (e.g., size, R&D intensity) commonly determined in quantitative research cannot be determined. However, in the phases of data calibration and analysis these characteristics can be considered in addition due to the small size of the sample. Generally, the authors are very familiar with the sample and have extensive case knowledge common to qualitative research, which is useful in the phases of data calibration as well as during the interpretation of the results.

## Data calibration

Data calibration is of paramount importance for the quality of QCA. The present study followed the technique suggested by Basurto and Speer [2] to transform qualitative interview information into fuzzy sets by identifying measures, anchor points, interview coding, summarizing data through classification, and assigning and revising fuzzy set values. The interview data were coded in MaxQDA software and summarized in Microsoft Excel. Subsequently, the data were calibrated. According to Ragin [1] “fuzzy sets […] are calibrated using external criteria, which in turn must follow from and conform the researcher's conceptualization, definition, and labeling of the set in question.” For those conditions measured against several indicators, fuzzy set memberships were calculated based on a system of qualitative and quantitative indicators and thresholds. The groundwork for the system of indicators is described in the *original article*, which describes the configurational theorizing and relevant indicators from the literature. When available, established literature thresholds were chosen. However, the thresholds were often derived from qualitative observations in the interview data.

### Calibration of investments

Table 3 details how investments were assessed indirectly based on a combined logic of indicator thresholds for the absolute and relative size of investments, the status of investment and technology readiness level (TRL), to ensure that the calibrated set sufficiently reflects the observed spectrum of commitments. Especially the differentiation between diverse investments and major investments required the analysis of combined indicators and multiple observations to separate those with a particularly high commitment from those that are “mostly” committed.

**Table 3:** Detailed calibration of investments in a four-value fuzzy set.

Indicators	Selection of cut-off points	Calibration of fuzzy set membership
Total aggregated investment in CCU (Million US$)	Cut-off point based on case knowledge: The cheapest demo plant costs US$ 20m; activities in EOR & CCS were excluded^1^ ▪ Major investments >US$ 20m ▪ No major investment <US$ 20m	**No investments (0 – fully out)** if: ▪ Total aggregated investments in CCU =0 (excluding EOR & CCS^1^) **Past investments, not continued (0.33 – mostly out)** if both: ▪ Total aggregated investments in CCU >0 ▪ Active status of investments =0 **Diverse investments (0.67 – mostly in)** if both: ▪ Total aggregated investments in CCU >0 ▪ Active status of investments =1 and not more than one of the following three sub-condition applies (sum ≤1.9): ▪ TRL level of at least one CCU activity is >7 (except CO_2_ capture^2^) ▪ Total aggregated investment in CCU ≥US$ 20m ▪ Ratio CCU/ total investments >4% **Major investments (demo plant) (1 – fully in)** if: ▪ Active status of investments = 1 and two or more of the following three sub-conditions apply (sum >1.9) ▪ TRL level of at least one CCU activity is >7 (except CO_2_ capture^2^) ▪ Total aggregated investments in CCU ≥US$ 20m ▪ Ratio CCU/ total investments >4%
Ratio of CCU/total investment (in%)	$\frac{Total\;aggregated\;investments\;in\;CCU\;p.a.}{R\&D\;Expenses\;2017}$ Cut-off point based on the analysis of the distribution of data set at 4%, in line with R&D intensity threshold: ▪ High share of CCU investments >4% ▪ Low share of CCU investments <4%	
Status of investments	▪ Not active / past =0 ▪ Active =1	
TRL level of CCU activity	Cut-off point based on TRL definitions: Technology demonstration = TRL 7 [24] ▪ TRL of all activities (except CO_2_ capture^2^) is <7 ▪ TRL of at least one activity (except CO_2_ capture^2^) is >7	

Notes

1 Enhanced oil recovery (EOR) and carbon capture and storage (CCS) were excluded since these technologies are beyond the scope of CCU in this study.

2 CO_2_ capture is excluded since it is an advanced and readily available technology which is not implemented only due to a lack of market.

### Calibration of profitability

Table 4 details how profitability was assessed indirectly based on logical combinations of the experts’ judgments about production costs and revenues.

**Table 4:** Detailed calibration of profitability in a four-value fuzzy set.

Indicators	Selection of cut-off points	Calibration of fuzzy set membership
Production cost	Cut-off points based on experts' qualitative statements on whether production costs are expected to: • increase • decrease • ambivalent outlook	**Increased profitability (1 – fully in)** if one of the following combinations applies: ▪ Revenue increases + decreased production cost ▪ Revenue increases + constant production cost ▪ Constant revenues + decreased production cost **Constant profitability (0.67 – mostly in)** if: ▪ Constant revenues & constant production costs **Ambivalent profitability outlook (0.33 – mostly out)** if one of the following combinations applies: ▪ Revenue increases + increased production cost ▪ Revenue decreases + decreased production cost ▪ Ambivalent production costs + constant / increasing revenues ▪ Ambivalent revenues outlook + constant / decreasing production cost **Decreased profitability (0 – fully out)** if one of the following combinations applies: ▪ Constant revenues + increased production costs ▪ Revenue decreases + increased production cost ▪ Revenue decreases + constant production cost ▪ Ambivalent production costs + decreasing revenues ▪ Ambivalent revenues outlook + increasing production cost
Revenues	Cut-off points based on experts' qualitative statements on whether revenues are expected to: • increase • decrease • ambivalent outlook	

### Calibration of intangible value (IV)

Relevant categories and indicators for measuring IV were derived from the literature, in particular Lev [25]. Table 5 details how IV was assessed as a continuous fuzzy set with the mean of the indicator groups patents, product & customer value, and public perception. Within the sub-indicator groups a median is calculated to level out outlier values from the interviews. Across the indicator groups a mean is chosen to weigh all three categories equally. The thresholds were partially derived from qualitative observations in the interview data, e.g., the number of patents per year.

**Table 5:** Detailed calibration of intangible value in a continuous fuzzy set.

Indicator group	Sub-indicators	Selection of cut-off points and calibration of sub-indicators	Calibration of fuzzy set membership
Patents *(IPR)*	Patents submitted and granted for CCU *(IPR)*	▪ Many patents (>10 p.a.) (1 – fully in) ▪ Some patents (0.67 – mostly in) ▪ Potential future patents (0.33 – mostly out) ▪ No patents (0 – fully out)	The IV fuzzy set is calculated as the mean of three indicator groups as follows: IV=mean(IPR,PCV,PP) (3) with PCV=median(PI,CS) (4) PP=median(SR,PR,SI,SR) (5) For interpretation of the resulting continuous fuzzy set, the thresholds are defined as follows: **Significant IV is created (1 – fully in)** **Some IV is created (0.67 – mostly in)** **Few or no significant IV is created (0.33– mostly out)** **IV is not created (0 – fully out)**
Product & customer value (*PCV)*	Product image improvements from CCU *(PI)*	▪ Major improvement (1 – fully in) ▪ Minor improvement (0.67 – mostly in) ▪ Unsure (0.33 – mostly out) ▪ No change (0 – fully out)	
	Customer satisfaction improvements from CCU *(CS)*		
Public Perception *(PP)*	CCU is communicated in sustainability reporting *(SR)*	▪ Many or significant (1 – fully in) ▪ Envisaged/soon (0.67 – mostly in) ▪ Possibly in the future (0.33 – mostly out) ▪ No change (0 – fully out)	
	Public relations improvement from CCU *(PR)*	▪ Major improvement (1 – fully in) ▪ Minor improvement (0.67 – mostly in) ▪ Unsure (0.33 – mostly out) ▪ No change (0 – fully out)	
	Stakeholder interest in CCU, e.g., investors, politicians, NGOs *(SI)*	▪ From many stakeholders or a significant interest (1 – fully in) ▪ Some selected or at local level (0.67 – mostly in) ▪ Possibly in the future (0.33 – mostly out) ▪ No (0 – fully out)	
	Stakeholder reactions to CCU *(ST)*	▪ Only / very positive reactions (1 – fully in) ▪ Generally positive, some mixed reactions (0.67 – mostly in) ▪ Neutral: neither positive nor negative (0.51 – at threshold but tolerable) ▪ Overall mixed reactions (0.33 – mostly out) ▪ Overall negative reactions (0 – fully out)	

### Calibration of policy conditions

The calibration of policy conditions was derived directly from the interview data. The statements of the experts on policies were calibrated to the degree they support or hinder CCU implementation as shown in Table 6.

**Table 6:** Calibration of policy conditions as a four-value fuzzy set.

Indicators	Calibration of fuzzy-set membership
Degree to which relevant regulations and policies hinder or support CCU, e.g. ▪ Emission Trading Scheme (ETS) ▪ Renewable Energy Directive (RED) ▪ Fuel Quality Directive (FQD)	Policies are largely supportive (1 – fully in) Policies are partially supportive, require updates (0.67– mostly in) Policies are overall unfavorable except in selected/local cases (0.33– mostly out) All relevant policies are unsupportive (0 – fully out)

### Calibration of progress

Table 7 details how, to calibrate progress, we assessed the expectations of the experts concerning growth and transformation. As suggested by De Block and Vis [3] a cluster analysis assessed the spectrum of combinations for growth and transformation. Naims and Eppinger [19] illustrates the observed clusters and their interpretation, firstly as transformation winners and opportunists who both expect to benefit from CCU, and secondly those that do not expect to benefit, including transformation underdogs, pessimists, and impact sceptics. Hence, we calibrated the outcome progress using formulae summarizing the defined clusters, as detailed in Table 7. Consequently, transformation winners are fully in the set, whereas impact sceptics are fully out of the set.

**Table 7:** Detailed calibration of progress as a five-value fuzzy set.

Indicators	Selection of cut-off points and calibration of sub-indicators	Calibration of fuzzy-set membership
Transformation *(T)*	The degree of expected transformation is calculated based on three indicators which are calibrated as present (1), partially present (0.67) or absent (0): ▪ Entrepreneurship *(EN)* ▪ Modernization of industry *(MI)* ▪ Industrial symbiosis *(IS)* Transformation is calibrated based on the sum of indicators ts=EN+MI+IS ▪ For ts=3 a strong transformation is expected (T=1) ▪ For 3>ts>1 a partial transformation is expected (T=0.67) ▪ For 1≥ts>0 the transformation outlook is ambivalent (T=0.33) ▪ For ts=0 no transformation is expected (T=0)	The calibration of progress is derived from the observed clusters of combinations of growth and transformation depicted in Naims and Eppinger [19]: **Transformation winners (1 – fully in)** Expect a strong transformation (T=1) and growth for the entire value chain or their own sector (G≥0.83) **Transformation opportunists (0.75 – mostly in)** Expect a partial transformation (T=0.67) but growth in their firm's sector (G=0.83) **Transformation underdogs (0.49 – below threshold)** Expect a strong or partial transformation (T≥0.33) but growth in other sectors (0.33≤G≤0.67) **Transformation pessimists (0.25 – mostly out)** Expect a strong transformation (T=1) but no growth at all (G=0) **Impact sceptics (0 – fully out)** Ambivalent about transformation impacts (T=0.33) and see no growth or for other sectors (G≤0.67)
Growth (*G)*	The degree of expected growth based on qualitative statements of the interviewed experts about economic growth of GDP, exports, employment, and local competitiveness is calibrated according to the following scale: ▪ Growth in the entire value chain (G=1) ▪ Growth in the firm's sector (G=0.83) ▪ Growth in other sectors (G=0.67) ▪ Ambivalent growth outlook (G=0.33) ▪ No growth expected (G=0)	

After completing the data matrix, all calibrations and thresholds were revised to improve their quality and consistency. The assigning of thresholds and degrees of set membership were made explicit, in accordance with the recommendations by De Block and Vis [3] for calibrating qualitative information. Through testing and revising with different thresholds, the calibrations were improved to allow for robust interpretations. Moreover, selected sensitivity checks revealed that small changes in the data assessment did not significantly impact the overall results of the analysis, since the formulae combine a multitude of indicators.

### Results: set membership of cases

The result of the data calibration is the set membership of all cases summarized in Table 8. While the raw data must remain confidential as agreed with the interviewed experts before the interviews, the calibrated data is anonymized and does not allow any identification on the individual cases.

**Table 8:** Set membership of cases.

Cases	Inv	Prof	IV	Pol	Prog
A	0.33	0.00	0.56	1.00	0.49
B	1.00	0.33	0.84	0.67	0.00
C	0.67	0.00	0.61	0.00	0.49
D	0.67	0.00	0.61	0.33	0.49
E	1.00	1.00	0.67	0.00	1.00
F	0.00	0.00	0.17	0.33	0.25
G	0.00	0.00	0.84	0.67	1.00
H	1.00	0.67	0.39	0.00	1.00
I	1.00	0.00	0.56	0.00	0.25
J	1.00	0.00	0.67	0.00	1.00
K	1.00	0.00	0.61	0.00	0.25
L	1.00	0.33	0.78	0.00	1.00
M	1.00	0.33	0.78	0.67	1.00
N	1.00	1.00	0.72	0.00	1.00
O	0.67	1.00	0.72	0.00	1.00
P	1.00	1.00	0.84	0.00	0.49
Q	1.00	1.00	0.78	1.00	1.00
R	1.00	1.00	0.78	0.67	1.00
S	1.00	1.00	0.89	1.00	0.75
T	1.00	1.00	0.89	1.00	0.75
U	0.67	0.33	0.59	0.00	0.49
V	0.67	0.33	0.59	0.33	0.00
W	0.33	0.00	0.78	0.00	0.49
X	0.67	0.00	0.67	0.00	0.75
Y	0.67	0.00	0.73	0.00	1.00

### Results: truth tables

Consequently, fs/QCA software was used to identify truth tables for the presence (Table 9) and the absence (Table 10) of the outcome from the calibrated data. The presented truth tables are hence the concluding result of data calibration of our empirical sample. Consequently, they permit the configurational analysis and interpretation described in Naims and Eppinger [19].

**Table 9:** Truth table for the presence of the outcome progress

Inv	Prod	IV	Pol	Cases	Prog	Consistency
1	0	1	0	10	1	0.75
1	1	1	0	4	1	0.81
1	1	1	1	4	1	0.78
0	0	1	1	2	1	0.81
1	0	1	1	2	0	0.57
0	0	0	0	1	1	0.75
1	1	0	0	1	1	0.84
0	0	1	0	1	1	0.84

**Table 10:** Truth table for the absence of the outcome progress

Inv	Prod	IV	Pol	Cases	∼Prog	Consistency
0	0	0	0	1	1	0.83
0	0	1	0	1	0	0.72
1	0	1	1	2	0	0.71
0	0	1	1	2	0	0.66
1	0	1	0	10	0	0.55
1	1	0	0	1	0	0.31
1	1	1	0	4	0	0.28
1	1	1	1	4	0	0.27

## Declaration of Competing Interests

The authors declare that they have no known competing financial interests or personal relationships that could have appeared to influence the work reported in this paper.
